# Pharmacological antagonism of interleukin-8 receptor CXCR2 inhibits inflammatory reactivity and is neuroprotective in an animal model of Alzheimer’s disease

**DOI:** 10.1186/s12974-015-0339-z

**Published:** 2015-08-09

**Authors:** Jae K Ryu, T Cho, Hyun B Choi, N Jantaratnotai, James G McLarnon

**Affiliations:** Department of Anesthesiology, Pharmacology and Therapeutics, University of British Columbia, 2176 Health Science Mall, Vancouver, British Columbia V6T 1Z3 Canada; Brain Research Centre, University of British Columbia, 2211 Wesbrook Mall, Vancouver, British Columbia Canada; Department of Pharmacology, Faculty of Science, Mahidol University, Bangkok, 10400 Thailand

**Keywords:** CXCR2, CXCR2 antagonist, SB332235, Interleukin-8 (IL-8), Amyloid-beta (Aβ_1–42_) intrahippocampal injection, Alzheimer’s disease (AD), AD animal model, Microgliosis, Neuroprotection, Oxidative damage

## Abstract

**Background:**

The chemokine interleukin-8 (IL-8) and its receptor CXCR2 contribute to chemotactic responses in Alzheimer’s disease (AD); however, properties of the ligand and receptor have not been characterized in animal models of disease. The primary aim of our study was to examine effects of pharmacological antagonism of CXCR2 as a strategy to inhibit receptor-mediated inflammatory reactivity and enhance neuronal viability in animals receiving intrahippocampal injection of amyloid-beta (Aβ_1–42_).

**Methods:**

In vivo studies used an animal model of Alzheimer’s disease incorporating injection of full-length Aβ_1–42_ into rat hippocampus. Immunohistochemical staining of rat brain was used to measure microgliosis, astrogliosis, neuronal viability, and oxidative stress. Western blot and Reverse Transcription PCR (RT-PCR) were used to determine levels of CXCR2 in animal tissue with the latter also used to determine expression of pro-inflammatory mediators. Immunostaining of human AD and non-demented (ND) tissue was also undertaken.

**Results:**

We initially determined that in the human brain, AD relative to ND tissue exhibited marked increases in expression of CXCR2 with cell-specific receptor expression prominent in microglia. In Aβ_1–42_-injected rat brain, CXCR2 and IL-8 showed time-dependent increases in expression, concomitant with enhanced gliosis, relative to controls phosphate-buffered saline (PBS) or reverse peptide Aβ_42–1_ injection. Administration of the competitive CXCR2 antagonist SB332235 to peptide-injected rats significantly reduced expression of CXCR2 and microgliosis, with astrogliosis unchanged. Double staining studies demonstrated localization of CXCR2 and microglial immunoreactivity nearby deposits of Aβ_1–42_ with SB332235 effective in inhibiting receptor expression and microgliosis. The numbers of neurons in granule cell layer (GCL) were reduced in rats receiving Aβ_1–42_, compared with PBS, with administration of SB332235 to peptide-injected animals conferring neuroprotection. Oxidative stress was indicated in the animal model since both 4-hydroxynonenal (4-HNE) and hydroethidine (HEt) were markedly elevated in Aβ_1–42_ vs PBS-injected rat brain and diminished with SB332235 treatment.

**Conclusion:**

Overall, the findings suggest critical roles for CXCR2-dependent inflammatory responses in an AD animal model with pharmacological modulation of the receptor effective in inhibiting inflammatory reactivity and conferring neuroprotection against oxidative damage.

## Background

Chronic inflammation is an inherent ongoing process in the progression of Alzheimer’s disease [1–3]. However, the specific mechanisms by which sustained inflammatory reactivity contributes to the progressive neuronal degeneration underlying loss of cognition in Alzheimer’s disease (AD) brain are not well understood. Some evidence suggests limited benefits of non-steroidal anti-inflammatory drugs (NSAIDS) [4] with the relatively small extent of drug efficacy attributed to previous deterioration in cognitive function in AD individuals prior to medication. Another possibility is that inflammatory reactivity in AD brain is manifest from activation of multiple pathways other than cyclooxygenase-dependent activity targeted by NSAIDS.

A critical component of inflammatory response is a chemokine-mediated mobilization of microglia in response to peptide deposition [3, 5–7]. A spectrum of chemokines contributes to inflammatory responses in disease [8, 9], with some evidence suggesting a prominent role for interleukin-8 (IL-8) in AD pathology. Gene microarray analysis has shown that IL-8 exhibits the largest increase in expression of any inflammatory factor in human microglia incubated with amyloid-beta (Aβ_1–42_) [10]. This same group also reported dose-dependent increases in production of IL-8 in human microglia stimulated with peptide [11]. Elevated cerebrospinal fluid (csf) levels of IL-8 have been documented in AD brain relative to controls [12]. Interestingly, IL-8 has been reported to potentiate Aβ_1–42_-induced expression and production of a number of pro-inflammatory cytokines in cultured human microglia [13].

Immunostaining for the IL-8 receptor CXCR2 has demonstrated receptor association with neuritic plaques in AD tissue [5, 14]. However, CXCR2 also ransduces IL-8-dependent cellular inflammatory chemokine responses in the periphery and brain. In the former case, the receptor is expressed by infiltrating neutrophils in chronic obstructive pulmonary disease (COPD) with inhibition of CXCR2-mediated inflammatory responses effective in attenuating lung damage [15]. Prominent CXCR2 activity in activated microglia has been reported in damaged brain with antagonism of receptor effective in reducing inflammation and promoting recovery in lesioned spinal cord [16], following traumatic brain insult [17] and in animal tumor models [18].

At present, pharmacological modulation of CXCR2 has not been examined in animal models of AD. We posited that given the high levels of IL-8 in AD brain that pharmacological inhibition of CXCR2 could serve as a novel strategy to protect neurons exposed to inflammatory microenvironments. To examine this hypothesis, we have used the compound SB332235, a selective inhibitor of CXCR2 in macrophage cells [15, 19], as a receptor antagonist to attenuate microglial inflammatory reactivity induced by Aβ_1–42_ intrahippocampal injection. Specifically, SB332235 has been examined in vivo as a modulator of CXCR2 cell-specific association, gliosis, microglial chemotactic response, oxidative stress factors, and neuronal viability.

## Methods

### Human brain tissue

#### Preparation of human ND and AD sections

The procedures used to isolate postmortem tissue have been described [20]. Entorhinal cortical sections from six ND cases (ages from 60 to 85 years, postmortem intervals, 6–24 h) and six AD cases (ages from 64 to 87 years, postmortem intervals, 5–10 h) were obtained from the Kinsmen Laboratory brain bank at the University of British Columbia (UBC, Vancouver, British Columbia, Canada). Average age of individuals and mean postmortem delay did not differ significantly between AD and ND cases. The ND cases exhibited no clinical or pathological history of dementia or other neurological disorders. Five of the ND cases were scored as Braak stage I with one case scored as Braak stage II [21]. All cases of AD met the clinical criteria and postmortem confirmation for AD [22] and were characterized by high levels of plaque density and neurofibrillary tangles. The AD cases were rated as Braak V (one case) or VI (five cases).

#### Immunohistochemical staining and analysis in human ND and AD sections

For immunofluorescent staining, free-floating sections (30 μm) from ND and AD tissues were washed in phosphate-buffered saline (PBS) with Triton X-100 (PBST; 0.01 M PBS, pH 7.4, containing 0.3 % Triton X-100) and transferred into 5 % skim milk in PBST for 1 h. Sections were then incubated for 48 h at 4 °C with antibodies for CXCR2, HLA-DR, or GFAP and then rinsed in PBST and incubated with Alexa Fluor 488-conjugated goat anti-rabbit IgG (1:200; Invitrogen) for 1 h at room temperature. After washing in PBST, sections were mounted on glass slides and coverslipped with Prolong Gold anti-fading agent (Invitrogen). For double-immunofluorescence staining [23], free-floating sections were incubated for 48 h at 4 °C with a mixture of two primary antibodies: CXCR2/HLA-DR and CXCR2/GFAP. After incubation with the indicated primary antibodies, sections were rinsed in PBST and incubated for 1 h at room temperature with a mixture of Alexa Fluor 488 goat anti-rabbit IgG (1:200; Invitrogen) and Alexa Fluor 594 goat anti-mouse IgG secondary antibody (1:200; Invitrogen).

#### In vivo studies using intrahippocampal injection of Aβ peptide

Surgical procedures. All animal procedures were approved by the UBC Animal Care Ethics Committee, with adherence to guidelines of the Canadian Council on Animal Care. Male Sprague Dawley rats (Charles River Laboratories, Montreal, QC, Canada) weighing 280–300 g were used for in vivo studies. In brief, rats were injected intraperitoneal (ip) with an anesthetic mixture of ketamine hydrochloride (100 mg/kg; Bimeda-MTC, Cambridge, ON, Canada) and xylazine hydrochloride (10 mg/kg; Bayer Inc., Etobicoke, ON, Canada) and were placed in a stereotaxic apparatus (David Kopf Instruments, Tujunga, CA, USA). Animals received stereotaxic injection of Aβ_1–42_ or controls (PBS or reverse peptide Aβ_42–1_) as previously described [6, 24–26]. Following skin incision to expose the skull, peptides (California Peptides, Napa, CA, USA) were slowly injected (0.2 μl/min) into the dentate gyrus region of rat hippocampus. Injection coordinates were as follows: anterior-posterior (AP), −3.3 mm; medial-lateral (ML), −1.6 mm; dorsoventral (DV), −3.2 mm; all measurements from bregma.

#### Preparation and administration of chemicals

*Amyloid peptide.* The procedures for preparation of amyloid-beta peptide for intrahippocampal injection have been described [6, 25, 26]. Full-length Aβ_1–42_ or reverse peptide Aβ_42–1_ (California Peptide, Napa, CA, USA) was first dissolved in 35 % acetonitrile (Sigma) and further diluted to 500 μM with incremental additions of PBS with vortexing. The peptide solution was subsequently incubated at 37 °C for 18 h to promote fibrillization and aggregation and stored at 20 °C [11, 24]. Peptides (2 nmol) were injected for durations of 1, 3, and 7 days in this work.

SB332235. This compound was kindly donated by GlaxoSmithKline (709 Swedeland Road, King of Prussia, PA, USA). The compound was dissolved in a saline solution and applied by ip injection at a single dose of 1 mg/kg at the time of peptide injection. SB332235 has been characterized as a specific antagonist for CXCR2-mediated functional responses [15, 27].

#### Immunohistochemical staining of rat brain

Animals were transcardially perfused with heparinized cold saline followed by 4 % paraformaldehyde under ketamine/xylazine anesthesia. Brains were then removed, postfixed, cryoprotected, and cut into 40-μm sections [6]. Free-floating sections were processed for immunohistochemistry as described previously [6, 24, 26]. Briefly, sections were incubated in PBS containing 1 % bovine serum albumin, normal goat serum (NGS), and 0.2 % Triton X-100 (Sigma-Aldrich, St Louis, MO, USA) for 1 h. Sections were incubated overnight at 4 °C with the following primary antibodies: anti-glial fibrillary acidic protein, a marker for astrocytes (GFAP; 1:1000; Sigma-Aldrich), anti-neuronal nuclei (NeuN; 1:500; Chemicon, Temecula, CA, USA), and two specific microglial antibodies (anti-ionized calcium-binding adapter molecule 1 (Iba-1; 1:500; Wako Chemicals, Richmond VA, USA) and HLA-DR (1:1000; Dako, Mississauga, ON, Canada). Other antibodies used included ones for CXCR2 (1:500; Santa Cruz Biotechnology, Santa Cruz, CA, USA), Aβ_1–42_ (1:100; Dako), and 4-hydroxynonenal (4-HNE, 1:500 Jaica, Shizuoka, Japan). Sections were rinsed in PBS with 0.5 % BSA and incubated with secondary antibodies conjugated with Alexa Fluor 488 or 594 (1:200; Invitrogen, Burlington, ON, Canada) for 1 h in the dark.

In this work, double immunostaining was also carried out for microglial and astrocytic CXCR2 expression. In the former case, since Iba-1 antibody was raised in rabbit, mouse OX-42 (1:500; Serotec, Oxford, UK) was used for staining of receptor in microglia. CXCR2 association with astrocytes used respective antibodies for receptor/cell of CXCR2/GFAP. Sections were rinsed in PBS with 0.5 % BSA and incubated with a mixture of secondary antibodies (Alexa Fluor 488 and 594; 1:100; Invitrogen).

To determine production of reactive oxygen species (ROS), peptide-injected animals received ip injection of 1 mg/kg hydroethidine (HEt; Molecular Probes) which is oxidized to ethidium bromide in the presence of superoxide radicals [28]. At 3 h following HEt injection, animals were killed by transcardiac saline perfusion and brains were removed and frozen. Coronal sections (40-μm thickness) of hippocampus were examined under a Zeiss Axioplan 2 fluorescent microscope equipped with an ethidium filter and digital video camera (DVC) system (Diagnostic Instruments, Sterling Heights, MI, USA).

#### Immunohistochemical analysis of rat brain

Quantification of immunohistochemical staining followed published procedures [25, 26, 29]. Digitized images were obtained with a Zeiss Axioplan 2 fluorescent microscope equipped with a DVC system. Quantitative image analysis for the immunostained rat hippocampal sections was performed on three equally spaced sections through the level of the injection site. In each stained section, hippocampal boundaries were outlined with the granule cell layer (GCL) denoted as the superior blade of dentate gyrus. The molecular layer (ML) was then defined as the region between GCL border and hippocampal fissure. Neuronal viability and lipid peroxidation were measured in GCL, and glial responses and superoxide production were measured in adjacent ML. Digitized images were analyzed using Northern Eclipse software (Empix Imaging, Mississauga, ON, Canada).

#### RT-PCR in peptide-injected rat hippocampus

The specific protocols for Reverse Transcription PCR (RT-PCR) closely followed those outlined in previous work from this laboratory [26, 29, 30]. Anesthetized animals were killed by decapitation at 1, 3, and 7 days after peptide injection. The control animals were killed at 3 days after PBS or reverse peptide Aβ_42–1_ injection. Brains were removed, and hippocampal tissues were freshly dissected onto cold metal tissue matrices (Harvard Apparatus) and quickly frozen in liquid nitrogen. Total RNA was extracted using Trizol reagent (Invitrogen) and processed using reverse transcriptase; cDNA products were amplified by PCR using a GeneAmp thermal cycler (Applied Biosystems, Foster City, CA, USA) with Taq polymerase. PCR primers (β-actin was used as a reaction control) were as follows: CXCR2: forward, 5′-GTC AGG ATC CAA GTT TAC CTC AAA AAT GG-3′; reverse, 5′-CTT AGG TCG ACG GTC TTA GAG AGT AGT GG-3′. The primers for IL-8 were as follows: forward, 5′-ACT GAG AGT GAT TGA GAG TGG AC AC-3′; reverse 5′-AAC CCT CTG CAC CCA GTT TTC-3′. Relative mRNA levels (stimulated values normalized to controls) were obtained using NIH ImageJ software 1.24 (National Institute of Health, Bethesda, MD, USA).

#### Western blot for CXCR2

Total protein from rat hippocampal tissue was used for Western blot analysis. Protein samples (50 μg) were subjected to SDS-PAGE prior to transfer onto a PVDF membrane (Millipore, Bedford, MA, USA), blocked with either 5 % skim milk or bovine serum albumin, and probed with anti-CXCR2 (1:200; Santa Cruz Biotechnology) and β-actin (1:5000; Abcam, Cambridge, MA, USA). HRP-conjugated secondary antibodies (GE Healthcare biosciences, Piscataway, NJ, USA) were used to develop immunoblots which were processed using enhanced chemiluminescence (ECL) detection (GE Healthcare Biosciences). Band intensities were quantified using ImageJ software (NIH).

#### Statistical analysis

Results are presented as mean ± SEM. The statistical analysis was performed using a one-way ANOVA, followed by the Student–Newman–Keuls multiple comparison test or Student’s *t* test (GraphPad Prism 3.0; Graph Pad) with significance level set at *p* < 0.05.

## Results

### CXCR2 expression in AD and ND brain sections

**Fig. 1:**
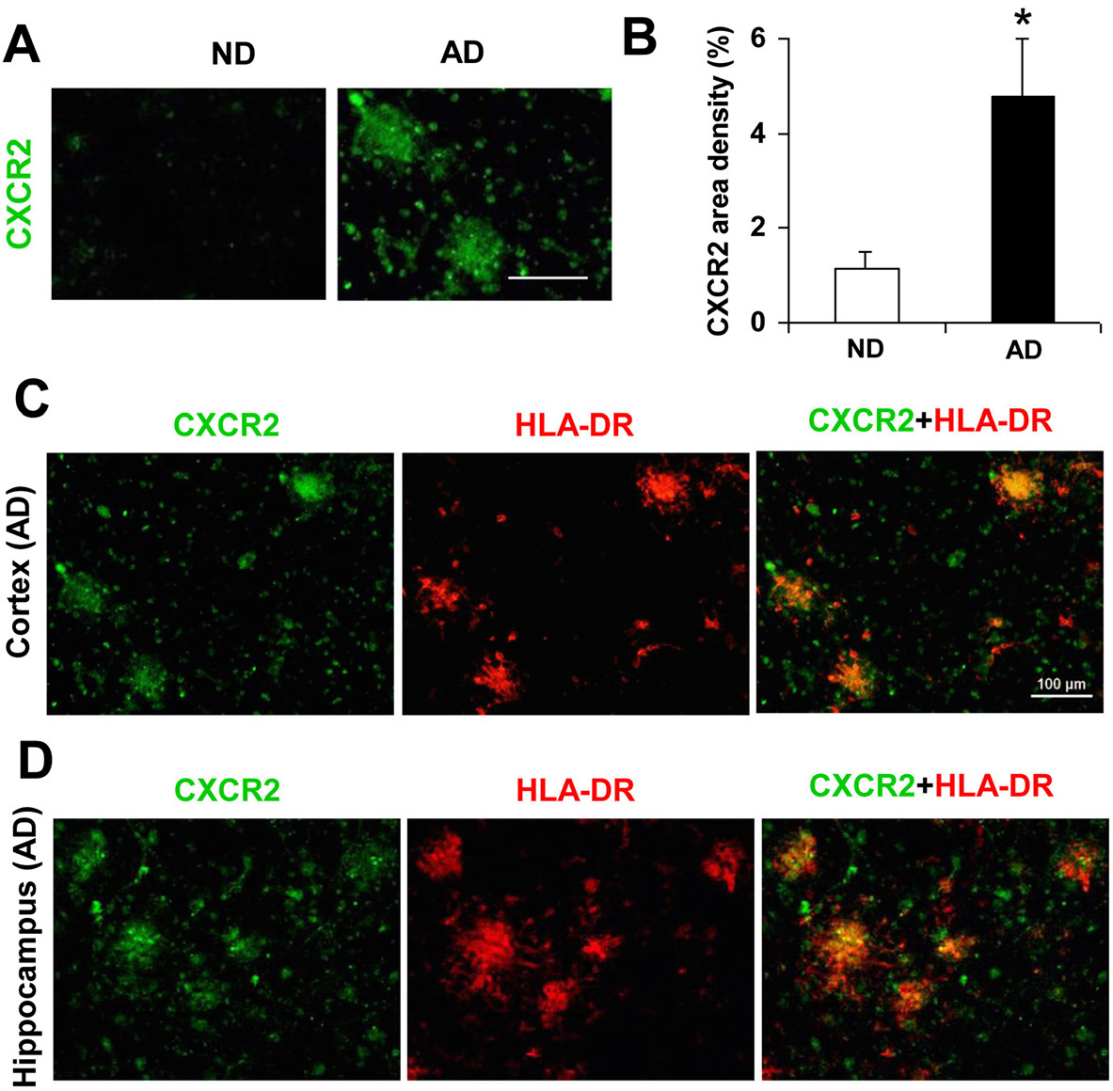
Staining patterns of CXCR2 in AD and ND cortical and hippocampal brain sections. **a** Representative CXCR2 immunoreactivity (ir) in cortical regions of ND and AD brain; *scale bar* represents 40 μm. **b** Quantification of CXCR2 area density in ND and AD sections (*N* = 6 cases for each); *asterisk* denotes *p* < 0.05. **c** Double staining of CXCR2 (*green*), HLA-DR-(+)ve microglia (*red*) and merged CXCR2/HLA-DR in cortical AD brain. **d** Double staining for the same markers in hippocampal brain sections; *scale bar* for **c** (and **d**, is same as **c**) is 100 μm

Brain tissue from AD and ND individuals was first analyzed for expression of CXCR2. Representative immunostaining demonstrated low levels of CXCR2 in areas of entorhinal cortex from ND tissue with a considerably elevated expression of the IL-8 receptor in AD sections (Fig. 1a). Quantification for CXCR2 expression is presented in Fig. 1b (*N* = 6 for each of AD/ND). The area density of CXCR2 was increased 4.4-fold in AD, compared with ND, brain tissue.

Since the focus of the study was pharmacological modulation of CXCR2-mediated inflammatory reactivity in vivo, we also examined receptor expression and the microglial marker HLA-DR in AD brain. Representative single and double staining for CXCR2/HLA-DR in cortical brain sections are presented in Fig. 1c. The results demonstrated considerable co-localization for the two markers, a similar finding was made in all AD cases. Typical expression of CXCR2/HLA-DR in AD hippocampal brain tissue is shown in Fig. 1d. Both markers showed a marked extent of co-localization throughout areas of hippocampus. Although hippocampal tissue was limited in availability, similar co-localization between receptor and HLA-DR was evident in sections from *N* = 3 other AD cases. Although we did not attempt quantification for overall merged staining for CXCR2/HLA-DR, the findings from AD cortical and hippocampal tissue implicated microglial CXCR2 as a putative inflammatory mediator in the progression of AD pathology. Experiments were then designed to examine effects of pharmacological antagonism of CXCR2 in animal brain.

### Time-dependent CXCR2 expression in vivo

An Aβ_1–42_ intrahippocampal-injection animal model [6, 26, 30, 31] was used to characterize glial reactivity and neuronal viability and their pharmacological modulations with the CXCR2 antagonist, SB332235. Initial experiments examined the expression of CXCR2 and a ligand for the receptor, IL-8 at different durations following peptide injection.

**Fig. 2:**
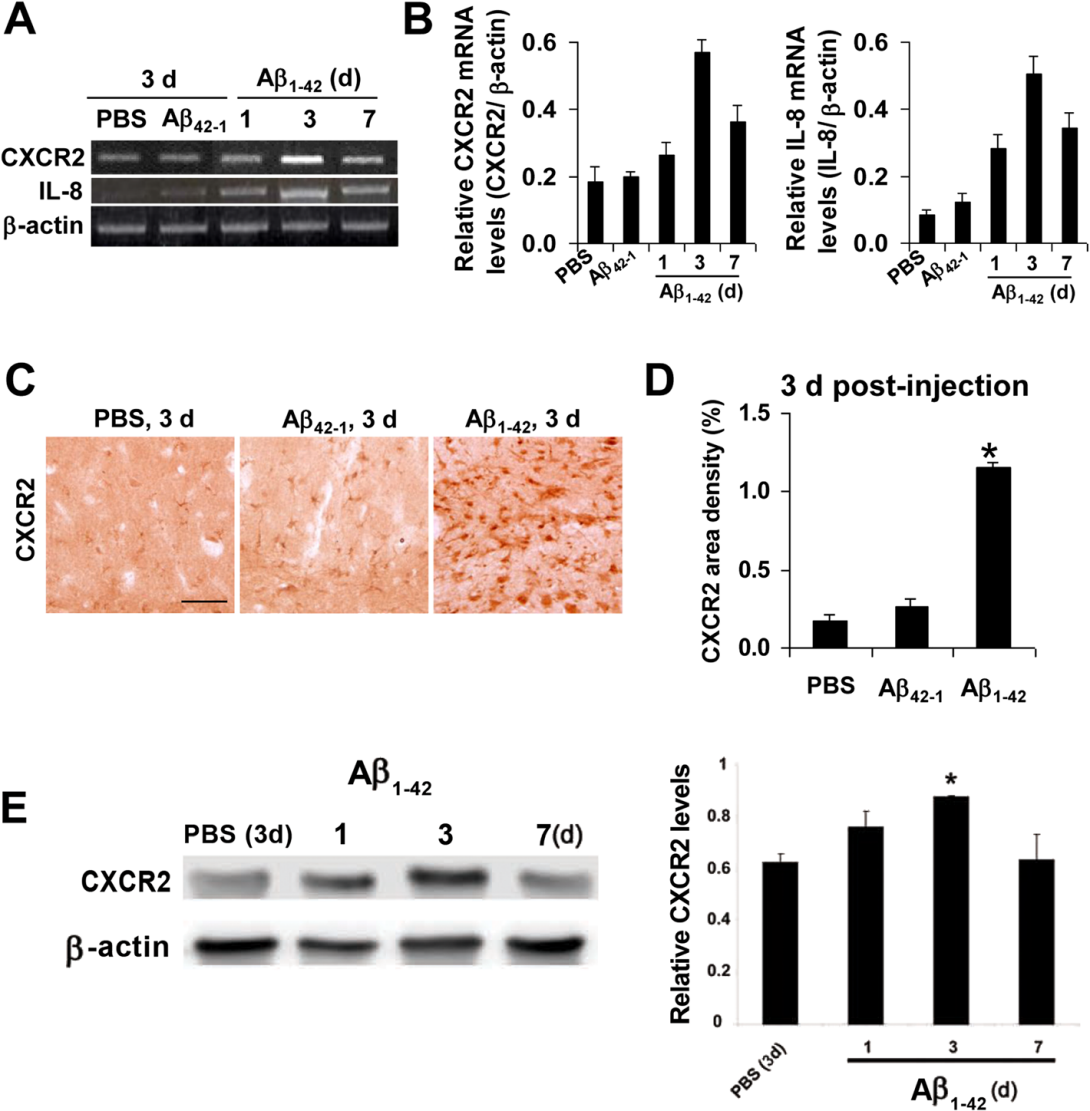
Expression of CXCR2 and IL-8 in ML region of rat dentate gyrus. **a** Representative RT-PCR for CXCR2 and IL-8 in controls (3 days post-injection of PBS or reverse peptide Aβ_42–1_) and in Aβ_1–42_-injected rat brain (1, 3, and 7 days post-injection); β-actin was used as a reaction standard. **b** Semi-quantification of RT-PCR for CXCR2 (*left bar graph*) and IL-8 (*right bar graph*); *N* = 5 animals per treatment group. **c** Typical CXCR2 ir for PBS, Aβ_42–1_, and Aβ_1–42_ (3 days post-injection); *scale bar* is for 70 μm. **d** Overall CXCR2 area density for the different animal groups (*N* = 4 animals per treatment group). *Asterisk* denotes *p* < 0.05 for Aβ_1–42_ vs PBS. **e** Representative Western blot for CXCR2 in control (3 days) and 1,3, and 7 days post-peptide injection. The *bar graph* shows relative CXCR2 levels for control and different durations of peptide injection (*N* = 4 animals per group)

Representative RT-PCR for CXCR2, and also for IL-8, is shown for durations of Aβ_1–42_ injections of 1, 3, and 7 days (Fig. 2a). Controls used both PBS and reverse peptide (Aβ_42–1_) with results shown at a single time point of 3 days post-injection. The results showed levels of both CXCR2 and IL-8 were higher at all time points post-Aβ_1–42_ injection compared with 3 days controls (PBS and Aβ_42–1_). Interestingly, both CXCR2 and ligand IL-8 were maximally expressed at 3 days following peptide injection.

Semi-quantification of data for CXCR2 expression (*N* = 5 animals/group) for the different peptide injection times is shown (Fig. 2b). Expression of CXCR2 (left bar graph) was increased with Aβ_1–42_ by respective amounts of 1.4-fold, 3.1-fold, and 1.9-fold (1, 3 and 7 days Aβ_1–42_) compared with 3 days PBS injection; the 3 and 7 days values representing significant increases. The corresponding data for IL-8 are presented in Fig. 2b (right bar graph) with expression of the chemokine (*N* = 5 animals/group) increased by 3.3-fold, 6-fold, and 4-fold with peptide injections (respective values for 1, 3, and 7 days application) compared with PBS 3 days injection; all values represent significant increases.

Additional experiments focused on CXCR2 expression in peptide-injected hippocampus. We used immunohistochemical staining in the ML of dentate gyrus to measure CXCR2 levels at the single time point of 3 days post-Aβ_1–42_ injection. As shown in Fig. 2c, relatively low levels of CXCR2 immunoreactivity (ir) were evident with either PBS (left panel) or reverse peptide (middle panel) controls. However, Aβ_1–42_-injected animals exhibited a marked increase in CXCR2 expression (right panel) with association of receptor in cells showing a glial morphology. Quantification of immunostaining data is presented in Fig. 2d (*N* = 5 animals/group). The area density of CXCR2 was considerably increased (by 6.3-fold) with Aβ_1–42_, compared with PBS, injection. Thus, upregulation of CXCR2 expression is a characteristic response to peptide injection in the AD animal model.

Western blot analysis was done to demonstrate protein expression of CXCR2. As shown in Fig. 2e, CXCR2 was minimally expressed in control, progressively increased at 1 and 3 days post-intrahippocampal injection of Aβ_1–42_ and returned to near control level at 7 days following peptide injection. Quantification of CXCR2 levels (*N* = 4 animals per group) showed that at 1 day post-Aβ_1–42_ injection, receptor expression was elevated but not significantly different from control. CXCR2 was maximally expressed at 3 days post-peptide and significantly increased (by 40 %) from levels with PBS injection.

### Cell-specific expression of CXCR2 in vivo

The patterns of CXCR2 immunostaining shown in Fig. 1 for human AD tissue indicated prominent receptor expression in microglia. Double immunostaining was used to study glial-dependent expression of CXCR2 in control and peptide-injected rat hippocampus. At maximal CXCR2 expression (3 days of Aβ_1–42_ injection), double staining was carried out to determine association of IL-8 receptor with microglia (OX-42 marker) and astrocytes (GFAP marker). As for previous studies on gliosis in the AD animal model, gliosis was measured in the ML region of the dentate gyrus. This procedure would serve to minimize contributions from receptor expression in neurons.

**Fig. 3:**
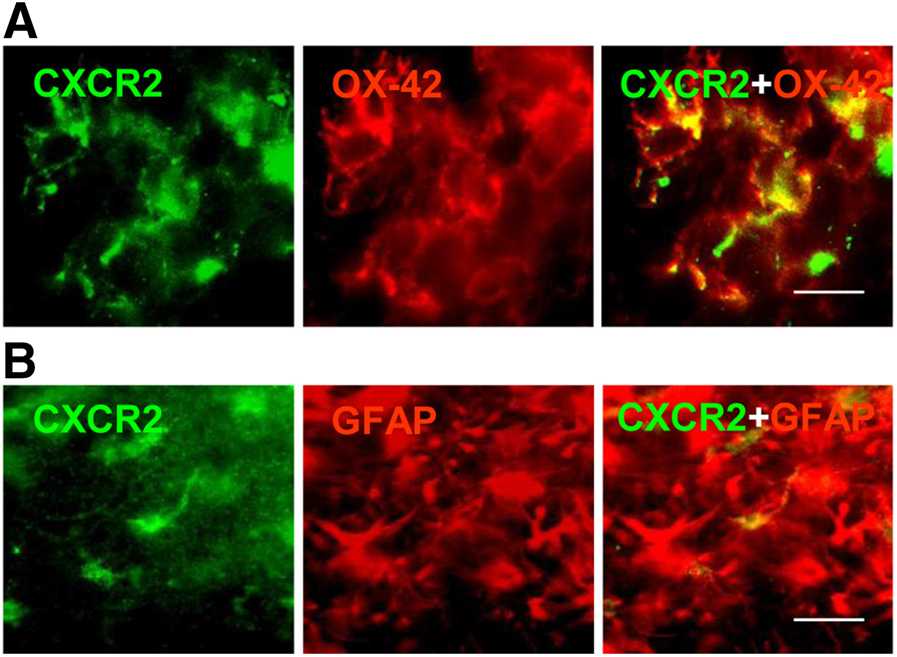
Cell-specific expression of CXCR2 in ML of dentate gyrus. **a** Representative single and merged staining of OX-42-(+)ve microglia with CXCR2 at 3 days post-Aβ_1–42_ intrahippocampal injection; *scale bar* is for 20 μm. **b** Single and merged staining of GFAP-(+)ve astrocytes with CXCR2 after 3 days of peptide injection; *scale bar* is for 15 μm

Representative patterns of immunostaining are shown for OX-42-(+)ve microglia and CXCR2 in Fig. 3a (3 days post-Aβ_1–42_ injection). Considerable association of the two markers was evident in the merged staining (right panel) with results indicating marked Aβ_1–42_ stimulation of microgliosis. Typical astrocytic staining (GFAP) with CXCR2 is presented in Fig. 3b. Merged staining indicated that a relatively low proportion of astrocytes were co-localized with CXCR2 (right panel, Fig. 3b).

The immunostaining results with both OX-42 and GFAP suggested considerable gliosis was induced in peptide-injected rat hippocampus which was examined as a target for pharmacological modulation of CXCR2 using the receptor antagonist, SB332235.

### Effects of CXCR2 antagonist SB332235 on gliosis

Initial experiments were designed to examine effects of CXCR2 antagonism, at a single time point of 3 days post-peptide injection, on microgliosis and astrogliosis. Sections were isolated from the ML region of hippocampus to minimize neuronal expression of receptor. Animal groups received PBS and reverse peptide controls, Aβ_1–42_, Aβ_1–42_ with SB332235 treatment and SB332235 alone.

**Fig. 4:**
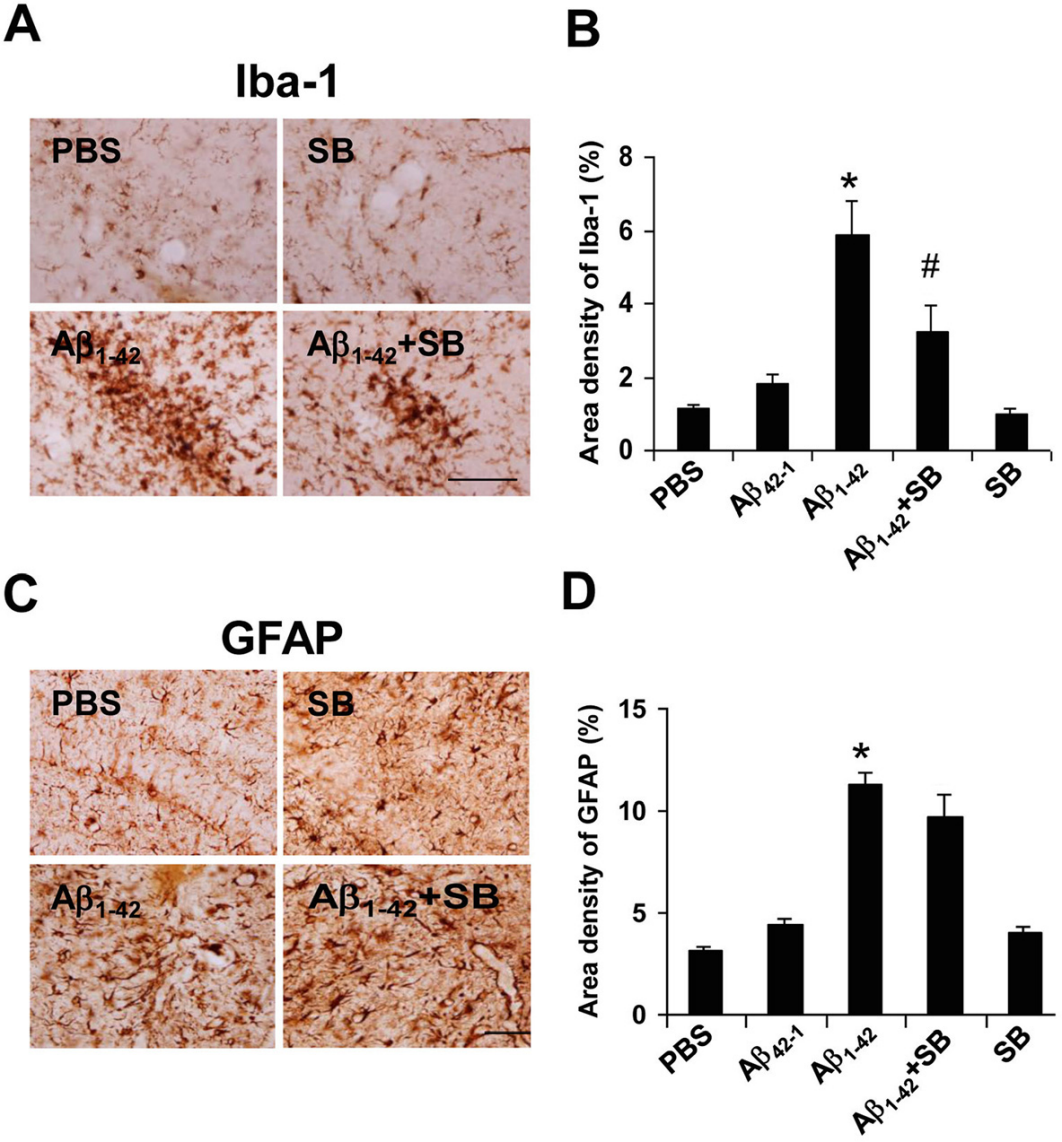
Effects of SB332235 on gliosis in ML region of dentate gyrus in peptide-injected hippocampus. **a** Representative microgliosis (Iba-1 marker) following 3 days injections with PBS (*upper left panel*), SB332235 alone (*upper right panel*), Aβ_1–42_ (*lower left panel*), and Aβ_1–42_ + SB332235 (*lower right panel*). **b** Overall area density for Iba-1 (*N* = 5 per treatment group). **c** Representative astrogliosis (GFAP marker) for PBS (*upper left panel*), SB332235 alone (*upper right panel*), Aβ_1–42_ (*lower left panel*), and Aβ_1–42_ + SB332235 (*lower right panel*). **d** Overall area density for GFAP (*N* = 5 per treatment group). *Scale bars* are for 80 μm. *p < 0.05 Aβ_1–42_ vs PBS; #*p* < 0.05 Aβ_1–42_ + SB332235 vs Aβ_1–42_

Representative microglial ir (Iba-1 marker) is shown in Fig. 4a and indicates relatively low numbers of cells in PBS control (upper left panel) and with SB332235 applied alone (upper right panel). Considerable microgliosis was induced following peptide injection (lower left panel). Treatment of peptide-injected animals with SB332235 was effective in attenuating microglial responses (lower right panel).

Quantification of data is presented in Fig. 4b (*N* = 5 animals/group) and also includes reverse peptide Aβ_42–1_ as a control animal group. Both PBS and reverse peptide demonstrated similar low values of microglial Iba-1 ir. Iba-1 ir, used as an index of microgliosis, was increased 3.5-fold in Aβ_1–42_, compared with PBS, injected rat brain. Peptide-administered animals receiving SB332235 exhibited significantly reduced microgliosis, by 45 %, compared with rats receiving Aβ_1–42_ in the absence of the CXCR2 antagonist. Similar levels of Iba-1 ir were measured with SB332235 administered alone and with PBS and Aβ_42–1_ controls.

Typical astrocytic staining (GFAP marker) indicated a considerably enhanced response in Aβ_1–42_-injected rats relative to PBS controls (Fig. 4c, left panels). Interestingly, unlike the results for microglia, astrogliosis remained elevated with SB332235 treatment of peptide-injected animals (lower right panel). GFAP staining with SB332235 applied separately (upper right panel) was similar to PBS control. Overall (*N* = 5 animals/group), peptide-injected hippocampus demonstrated an increased astrogliosis (by 3.5-fold) compared with PBS (Fig. 4d). In the presence of SB332235 application with Aβ_1–42_, GFAP ir was reduced by 14 %, an insignificant change compared with no drug treatment. Levels of GFAP were not significantly different between animals receiving SB332235 treatment and PBS or reverse peptide controls.

### Effects of SB332235 on CXCR2 expression, gliosis, and microglial chemotaxis nearby Aβ_1–42_ deposits

Previous work has indicated microglial chemotactic responses in the Aβ_1–42_ injection animal model as an initial inflammatory response to deposition of peptide [6, 7]. The consequence of this rapid response is the spatial localization of microgliosis and possibly upregulated CXCR2 ir in the vicinity of peptide deposits in the ML layer of dentate gyrus. Experiments were designed to examine chemotaxis in vivo and to measure SB332235 modulation of receptor expression and gliosis nearby amyloid deposits.

**Fig. 5:**
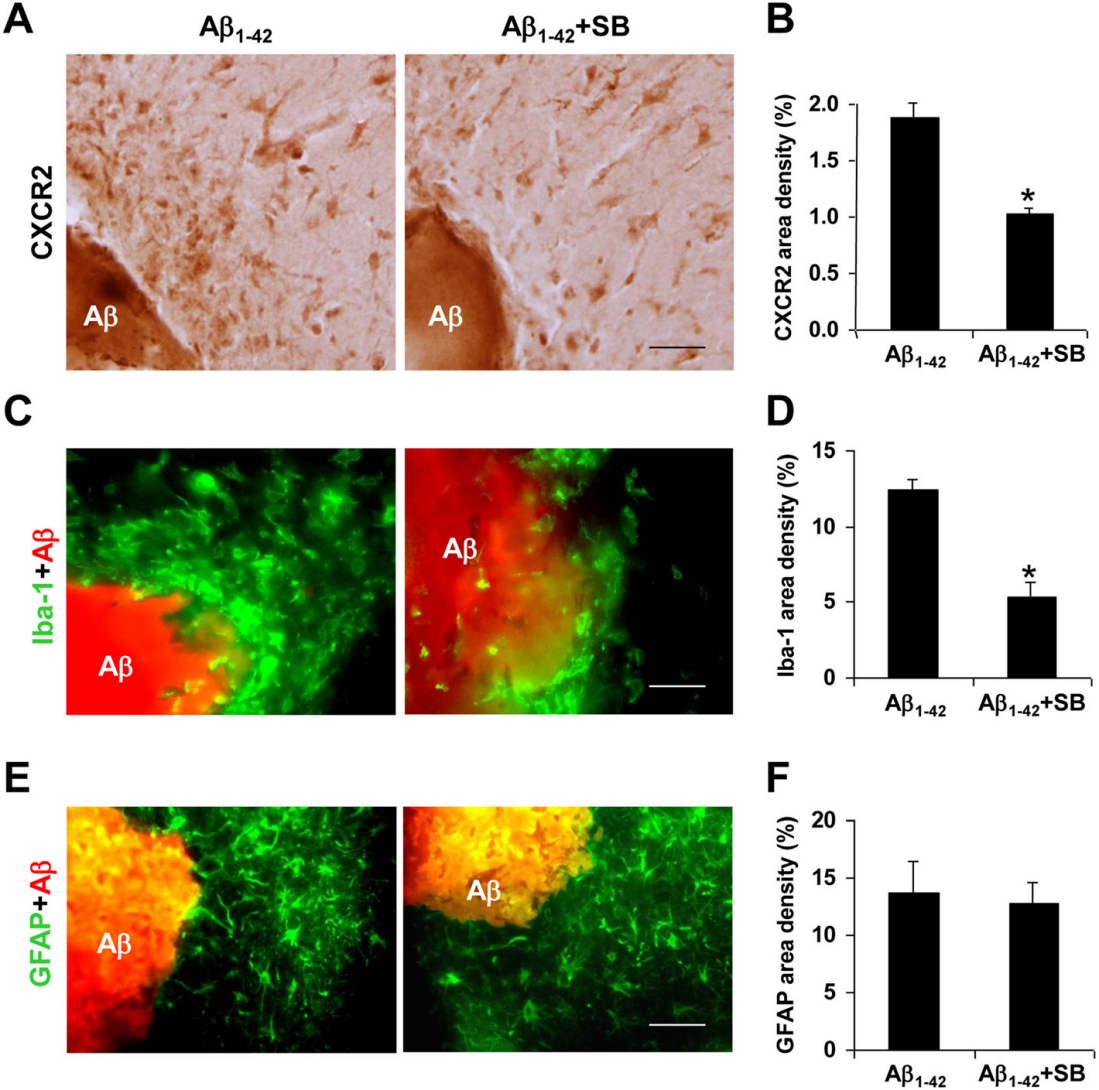
Effects of SB332235 on area density for CXCR2 and microglia and astrocyte responses in proximity to peptide deposits in ML region. **a** Representative CXCR2 ir nearby Aβ_1–42_ (3 days post-Aβ_1–42_ injection) in the absence (*left panel*) and presence (*right panel*) of SB332235 treatment; *scale bar* is for 50 μm. **b** Overall CXCR2 area density (*N* = 5 per group) in a single quadrant within 300 μm of peptide. **c** Representative Iba-1 ir nearby Aβ_1–42_ in the absence (*left panel*) and presence (*right panel*) of SB332235 treatment; *scale bar* is for 30 μm. **d** Overall Iba-1 area density (*N* = 5 per group) in regions within 300 μm of peptide. **e** Representative GFAP ir nearby peptide deposits in the absence (*left panel*) and presence of SB332235 (*right panel*); *scale bar* is for 30 μm. **f** Overall GFAP area density (*N* = 5 per group) within 300 μm of peptide. *p < 0.05 for Aβ_1–42_ vs Aβ_1–42_ + SB332235

We determined immunoreactivities of CXCR2, Iba-1, and GFAP within 300 μm of Aβ deposits in the ML region. The procedure defined quadrants of ML regions with a focal point denoted by Aβ plaque deposition. Representative staining for CXCR2 in proximity to peptide is shown in the absence (left column) and presence (right column) of SB332235 treatment of peptide-injected (3 days) animals (Fig. 5a). The distribution of CXCR2 ir was concentrated nearby Aβ deposits in peptide-injected hippocampus (left panel, Fig. 5a) with SB332235 effective in reducing receptor expression when administered with peptide (right panel, Fig. 5a). Overall (*N* = 5 animals/group), CXCR2 expression in proximity to Aβ was diminished by 45 % with application of SB332235 to peptide-injected animals (Fig. 5b).

Representative patterns of microglial expression (Iba-1 marker) are presented in Fig. 5c. Numbers of microglia were concentrated nearby Aβ then diminished with distance from deposits in both the absence (left panel) and presence (right panel) of SB332235 treatment of rats. Overall (*N* = 5 animals/group), SB332235 significantly inhibited Iba-1 ir by 57 % (Fig. 5d). The predominance of microglial staining in proximity to peptide suggested microglial chemotactic responses to Aβ_1–42_ deposits. Typical GFAP ir in Aβ_1–42_-injected animals, with and without SB332235 treatment, is presented in Fig. 5e. Similar homogenous distributions of astrocytic ir were evident in the presence, and absence, of CXCR2 receptor antagonist (left and right panels, Fig. 5e). Quantification of GFAP area density (*N* = 5 animals/group) is presented in Fig. 5f. The overall GFAP ir was not significantly different between untreated peptide-injected rats or animals receiving SB332235 application. In summary, microgliosis and CXCR2 area density are enhanced nearby Aβ with SB332235 effective in attenuating both responses.

### Effects of SB332235 on viability of GCL neurons and lipid peroxidation

#### Neuronal viability

**Fig. 6:**
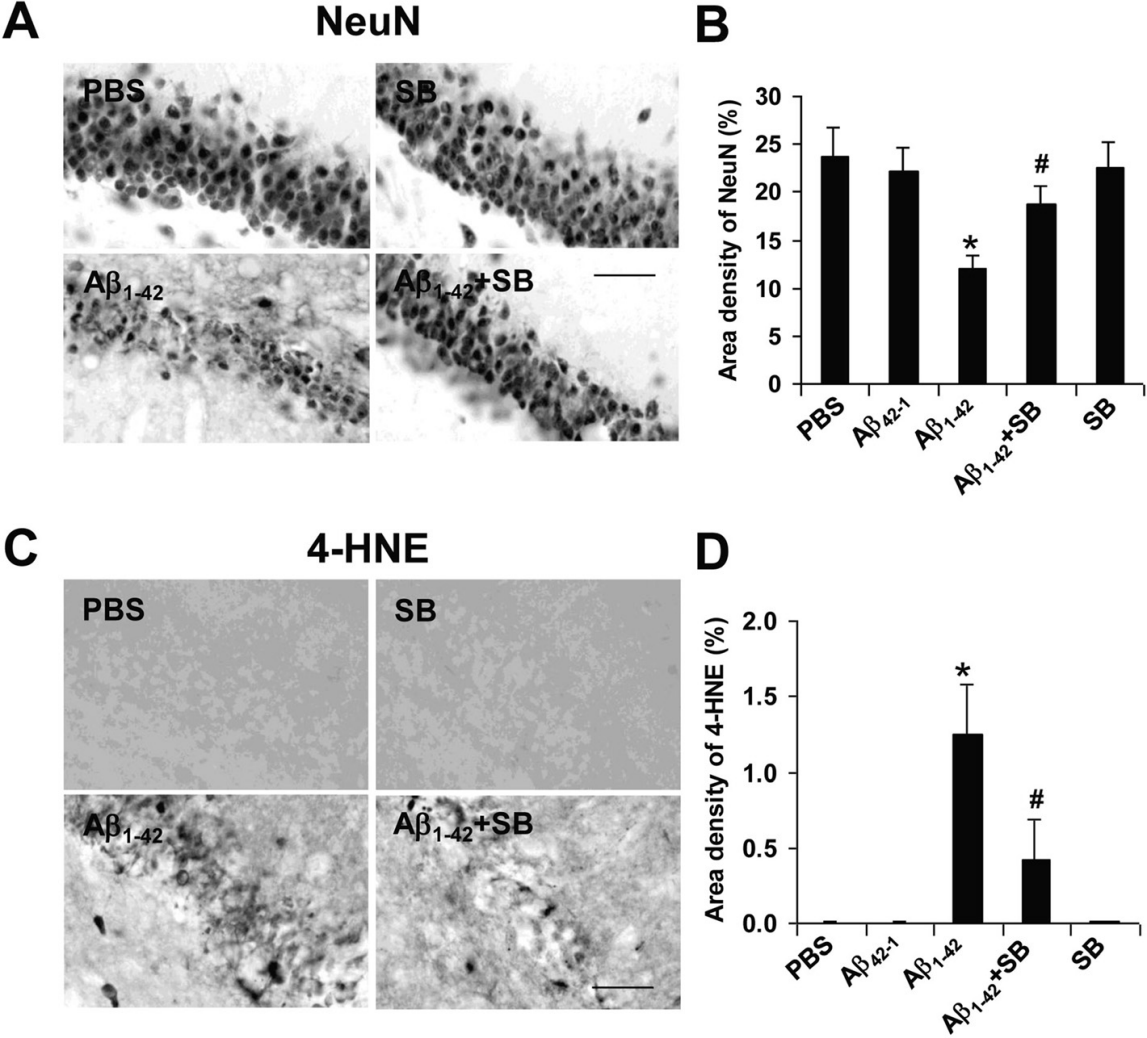
Neuroprotective and lipid peroxidation effects of SB332235 on GCL neurons. **a** Representative neuronal staining (NeuN) in PBS control (*upper left panel*), SB332235 alone (*upper right panel*), Aβ_1–42_ (*lower left panel*), and Aβ_1–42_ + SB332235 (*lower right panel*); results are for 3 days post-injection; *scale bar* represents 50 μm. **b** Area density of NeuN for the animal groups, *N* = 5 per group. *Asterisk* denotes *p* < 0.05. **c** Typical lipid peroxidation (4-HNE marker) levels following 3 days intrahippocampal injection of PBS (*upper left panel*), SB332235 alone (*upper right panel*), Aβ_1–42_ (*lower left panel*), and Aβ_1–42_ + SB332235 (*lower right panel*). *Scale bar* is for 50 μm. **d** Area density of 4-HNE for the different animal treatments, *N* = 5 per group. *p < 0.05 for Aβ_1–42_ vs PBS and #*p* < 0.05 for Aβ_1–42_ vs Aβ_1–42_ + SB332235

The neuroprotective efficacy of SB332235 administration to peptide-injected animals (3 days post-injection) was determined using NeuN as a marker for GCL neurons in dentate gyrus. Typical staining patterns for neurons for the different animal groups are presented in Fig. 6a. An intact GCL was evident in PBS control animals (upper left panel) or animals receiving reverse peptide, Aβ_42–1_ (not shown). Animals receiving intrahippocampal injection of Aβ_1–42_ exhibited a marked decrease of GCL neurons (lower left panel). The administration of SB332235 with peptide to animals markedly attenuated neuronal loss (lower right panel). Animals receiving SB332235 treatment in the absence of Aβ_1–42_ (upper right panel) showed similar patterns of NeuN staining as for PBS control.

Overall (*N* = 5 animals/group), Aβ_1–42_ injection caused a considerable loss of GCL neurons with levels of NeuN ir diminished by 56 % compared with PBS injection (Fig. 6b). However, SB332235 treatment conferred a significant degree of neuroprotection with numbers of neurons increased by 36 % compared to NeuN ir with peptide alone. Similar magnitudes of GCL neuron viability were determined for both controls (PBS and Aβ_42–1_) and SB332235 applied alone.

#### Lipid peroxidation

The relevance of oxidative stress and lipid peroxidation as a contributing factor to neuronal damage and cognitive deficiency has been indicated [32, 33]. We investigated if oxidative stress might be involved in neuronal damage in peptide-injected brain and if SB332235 could protect against oxidative-mediated activity. Experiments were designed to examine the overall changes in the lipid peroxidation product 4-hydroxynonenal (4-HNE) which is produced in cells under oxidative stress. These measurements were taken in the GCL region of dentate gyrus to relate oxidative effects to GCL neurons.

Typical staining patterns for 4-HNE are shown in Fig. 6c. Minimal 4-HNE ir was observed with PBS (upper left panel) or reverse peptide (data not shown) injections indicating a lack of oxidative damage in controls. However, considerable extents of 4-HNE staining were present in the Aβ_1–42_-injected rat hippocampus (lower left panel). Administration of SB332235 to peptide-injected animals was effective in reducing levels of lipid peroxidation product (lower right panel). The treatment of animals with SB332235 alone was without effect in induction of 4-HNE (upper right panel).

Quantification of data (*N* = 5 animals/group) indicated little or no measurable 4-HNE ir in PBS or reverse peptide controls whereas intrahippocampal peptide injection induced considerable lipid peroxidation product (Fig. 6d). Antagonism of CXCR2 with SB332235 reduced levels of 4-HNE (by 64 %) when applied to peptide-injected animals. Application of SB332235 alone had no effect on 4-HNE area density.

### Effects of SB332235 on superoxide

**Fig. 7:**
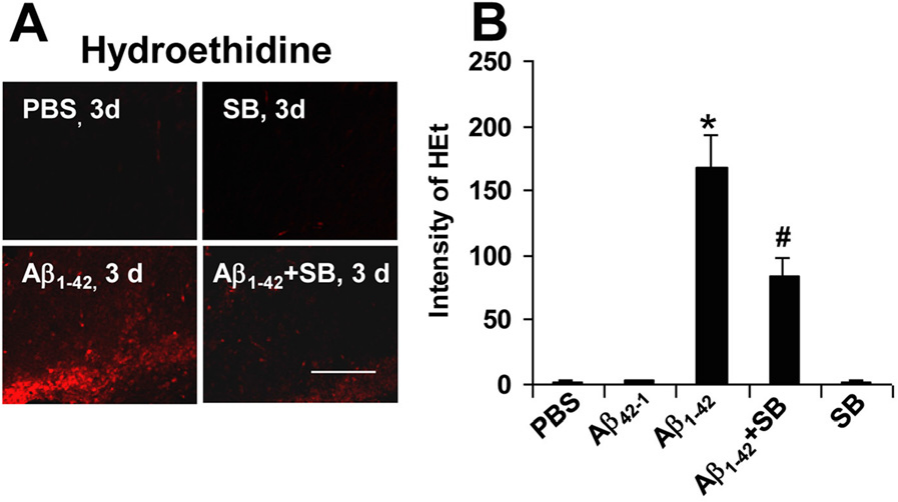
Effects of SB332235 on superoxide activity and inflammatory factors. **a** Representative superoxide ir (HEt) in region adjacent to GCL after 3 days intrahippocampal injection of PBS (*upper left panel*), SB332235 alone (*upper right panel*), Aβ_1–42_ (*lower left panel*), and Aβ_1–42_ + SB332235 (*lower right panel*); *scale bar* is for 120 μm. **b** Quantification of intensity of HEt for the different animal groups; *N* = 5 per group; *p < 0.05 for Aβ_1–42_ vs PBS and #*p* < 0.05 for Aβ_1–42_ vs Aβ_1–42_ + SB332235

A plethora of pro-inflammatory factors have been documented in AD brain [1]. Oxidative stress [34] and subsequent neuronal degeneration in peptide-injected inflamed brain could be mediated by superoxide production from activated microglia [35–37]. We used HEt as a cell-permeable probe to detect levels of superoxide adjacent to the GCL region. No evidence for HEt ir was found in PBS-injected animal brain (upper left panel, Fig. 7a) or in animals receiving reverse peptide injection (data not shown). Intrahippocampal Aβ_1–42_ injection caused a marked HEt ir (lower left panel) which was considerably attenuated with SB332235 treatment of peptide-injected animals (lower right panel). SB332235 administration alone produced minimal levels of superoxide (upper right panel).

The extent of HEt ir is shown in bar graphs (Fig. 7b) for the different animal treatments (*N* = 5 animal/group). Negligible HEt staining was evident for PBS and reverse peptide controls or for rats administered SB332235 alone. A high HEt ir was measured in Aβ_1–42_-injected rat brain with SB332235 treatment of peptide-injected animals significantly reducing levels of HEt ir by 55 %.

## Discussion

This study presents novel findings for enhanced expression of the chemokine IL-8 receptor CXCR2 in human AD brain and in ML region of dentate gyrus in Aβ_1–42_-injected rat hippocampus. Evidence is presented in the AD animal model indicating upregulation of CXCR2 may be linked with microglial-mediated responses which in turn are correlated with neuronal damage in inflamed brain. In essence, deposition of Aβ_1–42_ induces a microglial chemotactic response involving upregulation of CXCR2 and its ligand, IL-8. A net migration of microglia is manifest in clustering of cells in the vicinity of peptide leading to cell activation and subsequent production of an assemblage of pro-inflammatory mediators. Our findings suggest microglial-derived oxidative species and lipid peroxidation could contribute to oxidative stress damage to GCL neurons with pharmacological inhibition of CXCR2 efficacious in blocking inflammatory reactivity and attenuating neuronal damage.

The demonstration of upregulated CXCR2 in AD vs ND cortical brain tissue served as a rationale for the design of animal model experiments. Importantly, cortical brain tissue from AD individuals demonstrated areas of CXCR2 co-localization with activated microglia. Similar results were obtained in hippocampal brain sections in the few cases where tissue was available. The cell-specific association of CXCR2 supports the possibility that microglial-mediated inflammatory responses may be involved in AD pathology. Involvement of CXCR2 activation in inflamed brain is consistent with the finding that the receptor ligand, IL-8, is reported as the most highly upregulated factor from Aβ_1–42_-stimulated human microglia [10]. Interestingly, IL-8 priming of human microglia subsequently exposed to Aβ_1–42_ has been found to enhance cellular production of a host of inflammatory factors including pro-inflammatory cytokines [13].

The intrahippocampal injection of Aβ_1–42_ is an AD animal model characterized by enhanced inflammatory reactivity with pharmacological block of microgliosis correlated with increased viability of GCL neurons [25, 26, 30, 38]. In the present work, expression of CXCR2 and IL-8 showed similar time-dependent (1–7 days) increases following Aβ_1–42_, relative to controls (PBS and reverse peptide), intrahippocampal injection. Both receptor and ligand expressions were maximal at 3 days post-peptide injection and remained elevated at 7 days post-injection. Immunohistochemical staining exhibited similar results with Aβ_1–42_ injection yielding a fivefold increase in CXCR2 expression with Aβ_1–42_, relative to PBS, injection (time point of 3 days post-injection). Results from Western blot assay showed consistent trends in CXCR2 expression with duration of peptide injection with CXCR2 levels maximum at 3 days post-peptide injection.

At 3 days post-peptide injection, considerable extents of CXCR2 immunoreactivity were co-localized with microglia with lesser association of receptor with astrocytes (Fig. 3). Pharmacological antagonism of CXCR2 by SB332235 was examined with an initial focus on drug effects on gliosis at 3 days subsequent to intrahippocampal injection of Aβ_1–42_. A marked enhancement for both microgliosis and astrogliosis was evident in ML region of dentate gyrus compared with PBS or reverse peptide application (Fig. 4). Treatment of peptide-injected animals with SB332235 significantly inhibited microgliosis but was ineffective in attenuating astrogliosis. It can be noted that contributions from CXCR2-(+)ve neurons would be minimized in sections isolated from the ML region. In addition, the absence of myeloperoxidase (MPO) immunoreactivity (data not shown) indicated that CXCR2-mediated neutrophils did not contribute to inflammatory responses in Aβ_1–42_-injected rat brain.

Microglial chemotaxis is a rapid inflammatory response to Aβ deposition in the AD model [7, 26]. In this work, we measured net migration of microglia in a single quadrant in the immediate vicinity of peptide deposits in ML. Double staining was then used to determine CXCR2 and glial ir within 300 μm of Aβ_1–42_ deposits (Fig. 5). Animal treatment with SB332235 was examined for localized effects on CXCR2 area density and microgliosis and astrogliosis. Both CXCR2 area density and microglial ir were significantly attenuated by SB332235 administration with no effects of the compound on astrocytic responses. Although this component of study does not directly target chemotactic processes, the results suggest efficacy for SB332235 in inhibiting microglial responses and CXCR2-dependent activity nearby peptide.

Peptide-injected (3 days) rat brain exhibited a considerable loss of GCL neurons compared to PBS or reverse peptide injection (Fig. 6a, b). Treatment of Aβ_1–42_-injected rats with SB332235 conferred a significant degree of neuroprotection as shown by NeuN staining in the GCL region of dentate gyrus. Previous work using this animal model has demonstrated that drug actions which inhibit microgliosis are correlated with enhancement in numbers of GCL neurons [6, 26]. We also examined if lipid peroxidation could contribute to neurotoxicity by assessing 4-HNE ir in the GCL region. Overall, levels of 4-HNE were markedly elevated with Aβ_1–42_, and absent with PBS or Aβ_42–1_, intrahippocampal injection (all results obtained at 3 days post-injection). Animal treatment with SB332235 markedly inhibited 4-HNE levels in peptide-injected brain.

Previous work has demonstrated peptide-stimulated microglia as a prominent source of superoxide radical [35, 36]. Oxidative stress induced by superoxide species could be involved in the lipid peroxidation damage to neurons [34]. To examine this possibility, HEt ir was determined in the ML region of dentate gyrus. This region was chosen to correspond to the areas of microglial and astrocytic responses. Superoxide was not detectable in controls (PBS or Aβ_42–1_) at 3 days post-injection; however, considerable HEt ir was evident in Aβ_1–42_-injected brain. Treatment of peptide-injected animals with SB332235 was effective in attenuating levels of the superoxide marker. The neuroprotection conferred by SB332235 is consistent with previous results showing inhibition of microgliosis as a mechanism enhancing neuronal viability in the peptide-injected animal model but does not rule out possible direct effects of the CXCR2 antagonist on GCL neurons.

As noted above, direct intrahippocampal injection of Aβ_1–42_ serves as an AD animal model which exacerbates inflammatory reactivity. The model appears to be characterized as one in which an acute insult evolves into a chronic inflammatory perturbation in a relatively short time. The injection of peptide has particular utility in correlating effects of pharmacological modulation of microgliosis with viability of neurons. Validation of the model has been considered in terms of a comparison of cellular responses and processes with properties characteristic of AD brain tissue [39]. This comparison has shown similarities in a number of features including microglial and astroglial responses, abnormalities in microvasculature, and leakiness in BBB. Neuronal loss apparent in the AD model is the correlate of cognitive dysfunction in AD brain.

Our in vivo results provide evidence for efficacy of SB332235 at a time point associated with maximal expression for both CXCR2 and IL-8. However, the RT-PCR data suggest that both receptor and ligand expressions may remain elevated over longer times. In AD brain, expression of CXCR2 (Fig. 1) and IL-8 [12] is increased compared to levels in controls. In this case, the CXCR2 antagonist may have utility in reducing chronic inflammatory activity over extended times.

It is important to note that beneficial effects of microglial response and activation have been reported in AD brain [40–42]. Indeed, previous work on chemokine receptors in Tg 2576 mice has demonstrated that attenuation of Ccr2 in microglia was associated with abnormal accumulation of Aβ and increased mortality of animals [43]. Conversely, knockout of chemokine receptor Cx3cr1 was found to confer neuroprotection in a mouse model of AD [44]. Overall, a manifold of microglial-mediated inflammatory pathways is active in peptide-stimulated brain [1, 45] with diverging negative or positive effects on the viability of the neurovascular unit [46].

Our findings suggest the relevance in using transgenic animal models to examine pharmacological inhibition of CXCR2 as a strategy to enhance cognitive function. Such studies would reflect the effects of a progressive buildup of peptide deposits over time, rather than direct injection of amyloid, to more closely mimic chronic inflammation in AD brain. It should be emphasized that a number of chemokines, their receptors, and a host of non-chemokine factors could contribute to inflammatory reactivity in the progression of AD pathology. We suggest the merits in using a cocktail delivery of drugs as a strategy to examine effects for modulation of multiple components of chronic inflammation in treatment of the disease.

## Conclusion

Overall, this study has demonstrated competitive antagonism of CXCR2 as an effective strategy in attenuating chemokine receptor expression in microglia, the accumulation of microglia nearby peptide, and the cellular production of superoxide. The inhibition of a spectrum of inflammatory processes is correlated with an enhanced viability of granule cell neurons. Since CXCR2 and its ligand IL-8 are upregulated in AD, relative to ND, brain, modulation of CXCR2 represents a novel neuroprotective strategy to be tested in other AD animal models.
